# Minichromosome maintenance (Mcm) proteins, cyclin B1 and D1, phosphohistone H3 and *in situ* DNA replication for functional analysis of vulval intraepithelial neoplasia

**DOI:** 10.1038/sj.bjc.6600729

**Published:** 2003-01-28

**Authors:** E J Davidson, L S Morris, I S Scott, S M Rushbrook, K Bird, R A Laskey, G E Wilson, H C Kitchener, N Coleman, P L Stern

**Affiliations:** Immunology Group, Paterson Institute for Cancer Research, Christie Hospital NHS Trust Wilmslow Road, Manchester, M20 4BX, UK; MRC Cancer Cell Unit, Hutchison/MRC Research Centre Hills Road, Cambridge CB2 2XZ, UK; Department of Histopathology, St Mary's Hospital, Whitworth Park, Manchester M13 0JH, UK; Obstetrics and Gynaecology, St Mary's Hospital, Whitworth Park, Manchester M13 0JH, UK

**Keywords:** vulval intraepithelial neoplasia (VIN), minichromosome maintenance (Mcm) proteins, cyclin D1, cyclin B1, *in situ* DNA synthesis, phosphohistone H3, immunostaining

## Abstract

Vulval intraepithelial neoplasia (VIN) is defined histopathologically by distinctive abnormalities of cellular maturation and differentiation. To investigate the functional properties of VIN, the expression of several proteins involved in the regulation of the cell cycle as well as *in situ* DNA replication competence was analysed by immunohistochemistry. Snap-frozen vulval biopsies were graded as normal squamous epithelium (*n*=6), undifferentiated HPV positive VIN 1 (*n*=3), VIN 2 (*n*=8) and VIN 3 (*n*=20). Immunohistochemistry was performed using the following markers: cyclin D1 (expressed in middle/late G1), cyclin B1 (expressed in G2/early M), phosphorylated histone H3 (expressed during mitosis) and minichromosome maintenance (Mcm) proteins 2 and 5 (expressed during the cell cycle, but not in differentiated or quiescent cells). *In situ* DNA replication competence was used to identify S-phase cells. The percentage of positively stained nuclei in three representative microscopic fields was calculated per biopsy. In normal vulva, the expression of all markers was restricted to the proliferative compartment of the basal layer of the epithelium. In contrast in high-grade VIN, the majority of epithelial cells expressed the Mcm proteins from basal to superficial layer. The detection of cyclins B1 and D1, phospho-histone H3 and *in situ* DNA replication was also found through the full thickness of these lesions but by a lower proportion of the cells. This is consistent with these markers providing a series of ‘snapshots’ of the cell cycle status of individual cells. The low-grade VIN showed reduced expression of the cell cycle markers in relation to the level of dysplasia. The combination of these analyses establishes that the majority of VIN cells remain in a functional replicative or prereplicative state of the cell cycle. Clinical application of these analyses may provide a basis for improved diagnosis of VIN.

Vulval intraepithelial neoplasia (VIN) is a dysplastic condition of the squamous epithelium of the vulva (Buckley *et al*, 1984). It is characterised histopathologically by abnormalities of cellular maturation and differentiation. Mitotic figures, sometimes of abnormal or bizarre form, cellular and nuclear pleomorphism and cells with abnormally high nuclear to cytoplasmic ratios are observed throughout the various layers of the epithelium (Scully *et al*, 1994). Lesions are classified as VIN 1, VIN 2 and VIN 3 according to the proportion of cells from the basement membrane to the surface that are affected by these changes (Wilkinson *et al*, 1986). While the histopathological features associated with VIN are well described, however, little is currently understood about the functional properties of dysplastic vulval cells. One important factor is the association of undifferentiated VIN with high-risk human papillomavirus (HPV) infection (van Beurden *et al*, 1995). In HPV-infected epithelium, the viral oncogenes E6 and E7 drive the dysplasia by dysregulating the cell cycle checkpoints through interaction with the p53 and Rb regulatory proteins (Zur Hausen, 2002).

The cell cycle is the orchestrated series of molecular events, which coordinate the production of two daughter cells. It is divided into four distinct phases: G1, S, G2 and M. Progression through the successive stages of the cell cycle is accompanied by the alternate expression and lack of expression of specific regulatory proteins (Viallard *et al*, 2001). Dysregulation of the cell cycle is known to play an important role in the oncogenic transformation of cells (Johnson *et al*, 1995) and abnormal expression patterns of cell-cycle-associated cyclin D1 and retinoblastoma protein (Rb) have previously been documented in high-grade VIN lesions (Rolfe *et al*, 2001; Zamparelli *et al*, 2001). Other studies have demonstrated increased cellular proliferation in high-grade VIN using proliferation markers Ki-67 and proliferating cell nuclear antigen (PCNA) (van Hoeven and Kovatich, 1996; Modesitt *et al*, 2000). However, the scope of these analyses is restricted to high-grade VIN lesions and to the analysis of cell cycle proteins normally expressed during a single phase of the cell cycle (G1).

In the present study, we have investigated the expression of a number of cell cycle regulatory proteins and proliferation markers across the full spectrum of vulval dysplasia, from normal vulval epithelium to VIN 3. The approach used has been verified in a larger study of cell cycle kinetics in colorectal cancer (Scott *et al*, in submission). The markers used were cyclin D1, cyclin B1 and phosphorylated histone H3, all of which are normally expressed during distinct phases of the cell cycle (Viallard *et al*, 2001). Cyclin D1 is expressed in middle/late G1 and with CDK4/6 phosphorylates Rb, the inactivation of which is completed by cyclin E/CDK2 (Adams, 2001). Cyclin B1 is expressed in G2/early M and with CDK1 facilitates the processes involved in mitosis (Pines and Hunter, 1989; Ito, 2000; Taylor and Stark, 2001). Histone H3 is phosphorylated in mitosis and is associated with mitotic chromosome condensation (Mahadevan *et al*, 1991; Goto *et al*, 1999). To identify S-phase cells, *in situ* DNA replication competence was used. This technique has previously been validated in a variety of normal and neoplastic tissues, including normal squamous epithelium of the cervix and cervical intraepithelial neoplasia (CIN) 3 (Mills *et al*, 2000).

We have also compared the use of proliferation markers Mcm 2 and Mcm 5 in VIN. The Mcm proteins form an essential prereplicative complex by binding to DNA sites at which origin recognition complex and cdc6 proteins have sequentially bound. Mcm proteins are only present during the cell cycle and are lost in quiescent and differentiating cells (Ritzi *et al*, 1998). The use of an antibody directed against Mcm 5 has been shown to improve the specificity and sensitivity of cytological screening for CIN compared with the use of conventional cervical cytology alone (Freeman *et al*, 1999). Furthermore, antibodies against Mcm 2 and Mcm 5 have consistently been shown to identify a greater number of cycling cells than antibodies against Ki-67 or PCNA (Williams *et al*, 1998; Freeman *et al*, 1999). Here we report the expression pattern of Mcm proteins 2 and 5, cyclins D1 and B1, phosphohistone H3 and *in situ* DNA replication competence in normal squamous epithelium of the vulva, VIN 1, VIN 2 and VIN 3. The Mcm proteins identify all cells actively involved in the cell cycle, while cyclins D1 and B1, phosphohistone H3 and *in situ* DNA replication detect only a fraction of cells within a particular phase of the cell cycle.

## Materials and methods

### Patient material

In all, 37 vulval biopsies were obtained from women attending the Vulva Clinic at St Mary's Hospital in Manchester, UK. The Central Manchester Local Research Ethics Committee approved the study and all patients gave written informed consent. The tissue was coated in OCT and snap-frozen in liquid nitrogen. Seven and 10 *μ*m sections were cut onto aminopropyltriethoxysilane (APES)-coated slides and stained with haematoxylin and eosin (H/E) for histopathological analysis or stored at −70°C until required. Histological grading of all vulval specimens was performed by two independent histopathologists at St Mary's Hospital. Biopsies were graded as normal vulval epithelium (*n*=6), VIN 1 (*n*=3), VIN 2 (*n*=8) and VIN 3 (*n*=20). Only undifferentiated VIN associated with high-risk HPV infection as determined by PCR was included in this study. For HPV typing, DNA was extracted from the vulval biopsies by incubating the samples overnight at 37°C on a rotary mixer in 500 *μ*l of guanidinium isothyocyanate (GuScn) Lysis Buffer (12 g GuScn, 635 *μ*l 1 M sodium citrate, 425 *μ*l 30% sarcosyl, 50 *μ*l glycogen, 250 *μ*l 0.1 M DTT, up to 25.5 ml with double-distilled H_2_O). Samples were spun for 1 min and DNA was precipitated from the supernatant using isopropanol. The mixture was centrifuged at 13 000 rpm for 15 min, the pellet washed with 70% ethanol for 5 min and then dried at 56°C for 15 min before being dissolved in 400 *μ*l of double-distilled H_2_O. All specimens were positive for *β*-globin. HPV DNA was detected using GP5+/GP6+ consensus primers and genotyped using type specific primers for HPV 6/11, 16, 18, 31 and 33 (Van den Brule *et al*, 1990). Amplification products were resolved on 2% agarose gels and visualised by ethidium bromide staining.

### Immunohistochemical staining of specimens

Sections, 5-*μ*m, were quick-thawed and fixed in 4% paraformaldehyde in phosphate-buffered saline (PBS) for 5 min (Freeman *et al*, 1999). Endogenous peroxidase activity was quenched by incubation in 0.6% hydrogen peroxide in Tris-buffered saline (TBS) for 30 min. Sections were washed in TBS and blocked with 10% normal goat serum (DAKO, Ely, UK) and 10% bovine serum albumin (BSA) in TBS for 90 min at room temperature. The antibody directed against cyclin D1 was preabsorbed overnight at 4°C with 10% BSA in TBS. The antibodies against Mcm proteins 2 and 5, cyclin B1 and phospho-histone H3 did not require preabsorption. Final dilutions of primary antibody were prepared in 1% BSA and 0.1% Triton X-100 in TBS. In total, 50 *μ*l of antibody was added to each section and the slides were incubated at 4°C overnight in a humidified chamber. The following concentrations were found to produce optimal staining: Mcm 2 1 : 25, Mcm 5 1 : 25, cyclin D1 1 : 50, cyclin B1 1 : 600, phosphohistone H3 1 : 500. The primary antibody layer was omitted from a single section of each biopsy to serve as a negative control.

The slides were washed in 0.1% Tween 20 in TBS and incubated with biotinylated goat antimouse secondary antibody (Mcm 2 & 5 and cyclins B1 & D1) or biotinylated goat antirabbit secondary antibody (phosphohistone H3) (both from DAKO) at 1 : 500 in 1% BSA in TBS for 1 h at room temperature. Streptavidin horseradish peroxidase (HRP) (DAKO) was added for 30 min, followed by the substrate diaminobenzidine (DAB) for 10 min at room temperature and the reaction stopped with running water. The slides were counterstained with Harris' haematoxylin, dehydrated through graded ethanols and cleared in xylene. Coverslips were applied with DEPEX mounting medium (Gurr, BDH, Poole, Dorset, UK).

### *In situ* DNA replication competence

Frozen sections, 10 *μ*m, were quick-thawed, covered with 40 *μ*l incubation buffer (30 *μ*l SuNaSp/BSA with protease-inhibitor cocktail and 10 *μ*l 50 *μ*M Dig-dUTP nucleotide mix (Boehringer Mannheim)) and incubated at 37°C for 45 min in a humidified chamber. Slides were washed in PBS and fixed with 4% paraformaldehyde in PBS for 2 min (Mills *et al*, 2000). Endogenous peroxidase activity was quenched by incubation in 0.6% hydrogen peroxide in TBS for 3 min. Sections were coated with 50 *μ*l of rabbit anti-DIG/HRP antibody at 1 : 50 in 1% BSA in TBS and incubated at room temperature for 45 min. The slides were washed in TBS and DAB was used to develop the stain. The slides were counterstained with Harris' haematoxylin, dehydrated with ethanol and cleared in xylene. Coverslips were applied with DEPEX mounting medium.

### Scoring of stained sections

Scoring was carried out by two independent observers (EJD and PLS). The percentage of positively stained nuclei in three representative high-powered microscopic fields (approximately 1000 cells) was calculated per biopsy. In addition, the distribution of staining from basal layer to the surface layer of the epithelium was scored. Negative control sections from each biopsy showed negative immunostaining.

### Statistical analysis

The Spearman rank correlation coefficient (*r*_*s*_) was calculated for each marker across the spectrum of vulval dysplasia (normal, VIN 1, VIN 2 and VIN 3) using the raw data values from each patient. A value of *P<*0.05 was considered statistically significant.

## Results

### Expression of cell cycle markers and *in situ* DNA replication competence in normal squamous epithelium of the vulva

**Figure 1:**
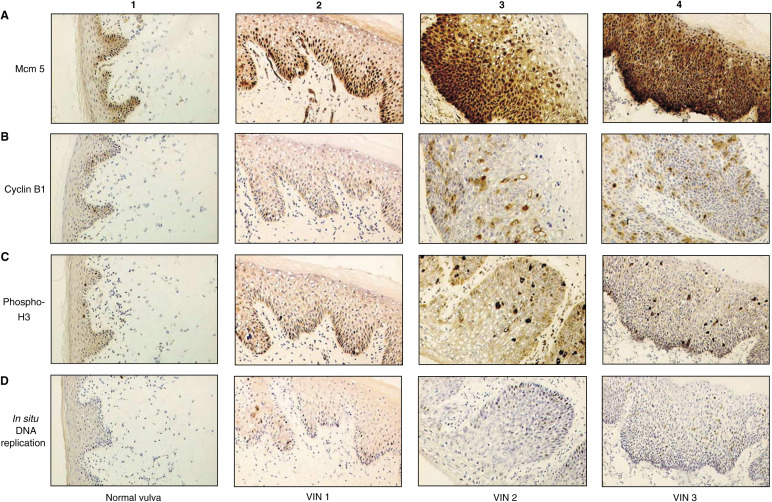
Immunostaining of normal vulval epithelium and VIN 1, VIN 2 and VIN 3 with cell cycle markers (x40): (**A1**) normal vulval epithelium shows that Mcm 5 expression is restricted to the basal epithelial layer; (**A2**) in VIN 1, >40% of cells express Mcm 5 in the basal and suprabasal epithelial layers; (**A3**) in VIN 2, >85% of cells express Mcm 5 and positive nuclei are detected in basal and superficial epithelial layers; (**A4**) VIN 3 shows >90% of cells express Mcm 5, extending throughout the full thickness of the epithelium; (**B1**) in normal vulval epithelium, expression of cyclin; (B1) is restricted to the basal epithelial layer. In VIN 1 (**B2**), VIN 2 (**B3**) and VIN 3 (**B4**), a greater proportion of cells express cyclin B1 compared with normal skin and positively stained nuclei are found in progressively more superficial epithelial layers, correlating with the level of dysplasia. **C1–4** and **D1–4** show a similar pattern of expression of phosphohistone H3 in normal vulva (**C1**), VIN 1 (**C2**), VIN 2 (**C3**) and VIN 3 (**C4**), and by *in situ* DNA replication in normal vulva (**D1**), VIN 1 (**D2**), VIN 2 (**D3**) and VIN 3 (**D4**).

In normal vulva, the expression of all cell cycle proteins and *in situ* DNA replication competence was restricted to the nuclei of the basal epithelial cells (Figure 1

). Differentiating cells in the more superficial layers of the epithelium were negative for all cell cycle markers. Mcm proteins 2 and 5 were found in the majority of cells in the basal epithelial layer (Table 1

**Table 1:** Percentage of cells expressing each cell cycle marker in normal vulval epithelium and VIN 1, VIN 2 and VIN 3

	**Normal (*n*=6)**	**VIN 1 (*n*=3)**	**VIN 2 (*n*=8)**	**VIN 3 (*n*=20)**	**Correlation coefficient**
Mcm 2	14.7 (9.9–19.4)	32.3 (19.2–52.5)	85.0 (80.5–94.1)	93.4 (82.9–97.8)	*r*_s_=+0.91
					*P<*0.001
Mcm 5	16.3 (9.7–21.7)	44.2 (33.6–61.0)	85.1 (79.6–91.7)	94.1 (84.5–98.9)	*r*_s_=+0.90
					*P<*0.001
Cyclin B1	1.9 (0.6–3.9)	2.5 (1.1–4.8)	9.5 (6.0–13.6)	9.7 (3.4–16.2)	*r*_s_=+0.68
					*P<*0.001
Phospho-H3	2.7 (0.5–4.7)	3.4 (1.0–7.3)	7.2 (1.8–14.1)	10.0 (3.0–26.2)	*r*_s_=+0.65
					*P<*0.001
*In situ* DNA replication	1.6 (1.8–3.4)	3.4 (2.0–5.3)	8.1 (4.7–12.3)	13.4 (5.5–30.2)	*r*_s_=+0.81
					*P<*0.001

This table shows the mean labelling indices (percentage of stained cells) for normal vulval epithelium and VIN 1, VIN 2 and VIN 3 with each of the cell cycle markers. Similar patterns of staining were observed for cyclins B1 and D1, but precise quantification of the latter was not possible. Ranges are given in parentheses. The proportion of positively stained nuclei increases with the level of dysplasia with each of the markers studied. The Spearman rank correlation coefficients and level of statistical significance are shown.

). In contrast, cells positively stained for cyclins B1, D1 and phosphohistone H3 and those exhibiting *in situ* DNA replication competence were found in a subset of the basal epithelial cell layer. Less than 5% of cells in the basal layer of the epithelium scored positive for cyclin D1 (data not shown).

### Expression of cell cycle markers and *in situ* DNA replication competence in VIN 1, VIN 2 and VIN 3

Positively stained nuclei were present in the superficial epithelial layers of all VIN lesions with antibodies against Mcm proteins 2 and 5, cyclin B1 and D1, phosphohistone H3 and by *in situ* DNA replication (Figure 1). The proportion of positively stained nuclei in the superficial epithelial layers correlated with the degree of dysplasia. The lowest proportion was found in the VIN 1 lesions, where positively stained nuclei were seen only in the suprabasal epithelial layers ( Table 1). A similar pattern of staining was seen for cyclins B1 and D1, but precise quantification of the latter was not possible because of cytoplasmic background staining.

In VIN 2 lesions, staining with all markers was seen in superficial layers of the epithelium and by a higher proportion of the cells overall compared with VIN 1. In VIN 3, full thickness staining of epithelial cells from basal to superficial layers was seen with all cell cycle markers. The majority of cells from the basement membrane to the surface of the epithelium expressed Mcm proteins 2 (93.4%) and 5 (94.1%). The detection of cyclins B1 and D1, phosphohistone H3 and *in situ* DNA replication was also found through the full thickness of these lesions, but by a lower proportion of cells.

There was a statistically significant correlation between the level of dysplasia and the proportion of positively stained nuclei with every marker investigated (Table 1). The highest Spearman rank correlation coefficients were observed for Mcm proteins 2 (*r*_s_=+0.91) and 5 (*r*_s_=+0.90), *P<*0.001. The correlation coefficients for cyclin B1 (*r*_s_=+0.68), phosphohistone H3 (*r*_s_=+0.65) and *in situ* DNA replication (*r*_s_=+0.81) were also statistically significant at the *P<*0.001 level.

## Discussion

In this study we have analysed the expression patterns of several cell cycle regulatory proteins and proliferation markers across the spectrum of vulval dysplasia, from normal squamous epithelium to VIN 3. The expression of all cell cycle markers and *in situ* DNA replication competence was restricted to the proliferative compartment of the basal layer of normal vulval squamous epithelium. In contrast, the majority of cells from basal to superficial layer expressed Mcm proteins 2 and 5 in VIN 3. Cyclin D1, B1, phosphohistone H3 and *in situ* DNA replication were also seen through the full thickness of VIN 3 lesions, but in a lower proportion of the cells. This is consistent with each marker providing a series of ‘snapshots’ of the cell cycle status of individual cells within the lesion. In low-grade VIN, reduced expression of all markers was seen in relation to the level of dysplasia, with positively stained nuclei found in the suprabasal layers of the epithelium in VIN 1 and extending into more superficial layers of the epithelium in VIN 2. There was a statistically significant correlation between an increasing level of dysplasia and a higher proportion of positively stained cells with each of the markers tested. The best markers of increasing dysplasia were the Mcm proteins 2 and 5, which gave correlation coefficients of *r*_s_=+0.91 and +0.90, respectively. Cyclin D1 expression was similar to that observed for cyclin B1, but was more difficult to quantify precisely because of cytoplasmic background staining.

The proportion of cells positive for Mcm proteins 2 and 5 was similar to that previously observed in normal squamous epithelium of the cervix and in low- and high-grade CIN (Williams *et al*, 1998). We also found that the proportion of cells demonstrating *in situ* DNA replication competence was consistent with that previously observed in the cervix (Mills *et al*, 2000). It should be noted, however, that those women who had ‘normal vulval epithelium’ by histology had presented with symptoms which necessitated a vulval biopsy. Whether this tissue is functionally normal is not known, but at least in histopathological terms, there was no evidence of VIN in these sections.

This study establishes that the majority of VIN cells remain in a functional replicative or prereplicative state of the cell cycle. All cells actively involved in the cell cycle are detected with antibodies against the Mcm proteins and a subset of these with each additional cell cycle marker. As a consequence of this, immunostaining of VIN lesions, particularly with antibodies against the Mcm proteins, may have a number of clinical applications. VIN 3 is a chronic condition with a significant risk of progression to invasive vulval carcinoma (Jones, 1995). Conventional therapy, including laser and surgical excision, is typically unsatisfactory since lesions are multifocal and show high rates of recurrence (Modesitt *et al*, 1998). Histological grading of VIN based on H/E sections does not always accurately distinguish between VIN 2 and VIN 3 (van Beurden *et al*, 1999). This may have significant clinical implications since the decision to treat rather than continue with conservative management may critically depend on this distinction (van Beurden *et al*, 1998). The application of these approaches may therefore provide a means of improved diagnosis of high-grade VIN.

In cervical dysplasia, persistent infection with high-risk HPV types potentiates the outgrowth of cervical intraepithelial neoplasia (CIN) by the inactivation of pRb and p53 by high-risk HPV E7 and E6, respectively. Since pRb inhibits transcription of the cyclin-dependent kinase inhibitor p16^INK4a^, p16 is markedly overexpressed in HPV-associated CIN and cervical cancers (Klaes *et al*, 2001). It is therefore possible that p16^INK4a^ may be a useful marker for dysplasia in VIN. Contrary to this expectation, Chan *et al*, (1998) claimed that the proportion of lesions with either undetectable p16 and/or pRb increased from benign lesions, through increasing grade of VIN to squamous carcinoma of the vulva. The latter study did not investigate the HPV status of the lesions and given the pathobiological differences between HPV positive and negative cancers (Crum, 1992), the precise relation of VIN and p16 status is still unclear. Inactivation of p16 has previously been shown to confer a loss of tumour-suppressor function in high-grade gliomas (Nishikawa *et al*, 1995). Another investigation of the inactivation of p16^INK4a^ and 14-3-3*σ* suggested the latter as early events in vulval squamous neoplasia (Gacso *et al*, 2002). Downregulation of 14-3-3*σ* in keratinocytes allows escape from replicative senescence and this is also accompanied by loss of p16^INK4a^. It has been shown that loss of 14-3-3*σ*, via methylation-dependent transcriptional silencing, occurs commonly in vulval neoplasia, and that it frequently coexists with silencing of p16 early in the natural history of disease. A closer examination of these data shows that most HPV-16-positive VIN 2/3 (11/12) do not have methylated p16^INK4a^, whereas in HPV-negative lesions, 8/11 VIN 3, 2/5 VIN 2 and 0/4 VIN 1 show p16 methylation. It is obvious that there is heterogeneity in the mechanisms underlying VIN in respect of viral and other influences on p16. The expression of Mcm proteins is a measure of licence for the cell cycle, which is independent of the consequences, or presence of HPV. Thus, it seems likely that combinations of these approaches will provide a useful synergism in the study of VIN. Mcm staining may also be useful in directing laser capture analysis of expressed transcripts encoding relevant viral and immunological factors in the ongoing biological analysis of VIN (Wong *et al*, 2000). Further work in long-term follow-up studies is required to establish the clinical relevance of these analyses.

## References

[bib1] Adams PD (2001) Regulation of the retinoblastoma tumor suppressor protein by cyclin/cdks. Biochim Biophys Acta 1471(3): M123–M133 11250068 10.1016/s0304-419x(01)00019-1

[bib2] Buckley CH, Butler EB, Fox H (1984) Vulvar intraepithelial neoplasia and microinvasive carcinoma of the vulva. J Clin Pathol 37: 1201–1211 6389601 10.1136/jcp.37.11.1201 PMC498984

[bib3] Chan MK, Cheung TH, Chung TK, Bao SY, Zhao CL, Nobori T, Wong YF (1998) Expression of p16INK4 and retinoblastoma protein Rb in vulvar lesions of Chinese women. Gynecol Oncol 68(2): 156–161 9514803 10.1006/gyno.1997.4914

[bib4] Crum CP (1992) Carcinoma of the vulva: epidemiology and pathogenesis. Obstet Gynecol 79: 448–454 1310806 10.1097/00006250-199203000-00025

[bib5] Freeman A, Morris LS, Mills AD, Stoeber K, Laskey RA, Williams GH, Coleman N (1999) Minichromosome maintenance proteins as biological markers of dysplasia and malignancy. Clin Cancer Res 5: 2121–2132 10473096

[bib6] Gacso M, Sullivan A, Repellin C, Brooks L, Farrell PJ, Tidy JA, Dunne B, Gusterson B, Evans DJ, Crook T (2002) Coincident inactivation of 14.3.3σ and p16^INK4A^ is an early event in vulval squamous neoplasia. Oncogene 21: 1876–1881 11896620 10.1038/sj.onc.1205256

[bib7] Goto H, Tomono Y, Ajiro K, Kosako H, Fujita M, Sakurai M, Okawa K, Iwamatsu A, Okigaki T, Takahashi T, Inagaki M (1999) Identification of a novel phosphorylation site on histone H3 coupled with mitotic chromosome condensation. J Biol Chem 274(36): 25543–25549 10464286 10.1074/jbc.274.36.25543

[bib8] Ito M (2000) Factors controlling cyclin B expression. Plant Mol Biol 43(5–6): 677–690 11089869 10.1023/a:1006336005587

[bib9] Johnson DG, Richie E, Conti C (1995) The cell cycle and cancer. Cancer Bull 47: 480–485

[bib10] Jones RW (1995) The natural history of vulvar intraepithelial neoplasia. Br J Obstet Gynaecol 102: 764–766 7547730 10.1111/j.1471-0528.1995.tb10839.x

[bib11] Klaes R, Friedrich T, Spitkovsky D, Ridder R, Rudy W, Petry U, Dallenbach-Hellweg G, Schimdt D, von Knebel Doeberitz M (2001) Overexpression pf p16(INK4A) as a specific marker for dyplastic and neoplastic epithelial cells of the cervix uteri. Int J Cancer 92: 276–284 11291057 10.1002/ijc.1174

[bib12] Mahadevan LC, Willis AC, Barratt MJ (1991) Rapid histone H3 phosphorylation in response to growth factors, phorbol esters, okadaic acid and protein synthesis inhibitors. Cell 65: 775–783 2040014 10.1016/0092-8674(91)90385-c

[bib13] Mills AD, Coleman N, Morris LS, Laskey RA (2000) Detection of S-phase cells in tissue sections by *in situ* DNA replication. Nat Cell Biol 2: 244–245 10783244 10.1038/35008670

[bib14] Modesitt SC, Groben PA, Walton LA, Fowler WCJ, Van Le L (2000) Expression of Ki-67 in vulvar carcinoma and vulvar intraepithelial neoplasia III: correlation with clinical prognostic factors. Gynecol Oncol 76(1): 51–55 10620441 10.1006/gyno.1999.5655

[bib15] Modesitt SC, Waters AB, Walton L, Fowler WC, Van Le L (1998) Vulvar intraepithelial neoplasia III: occult cancer and the impact of margin status on recurrence. Obstet Gynecol 92: 962–966 9840558 10.1016/s0029-7844(98)00350-0

[bib16] Nishikawa R, Furnari FB, Lin H, Arap W, Berger MS, Cavennee WK (1995) Loss of p16^INK4A^ expression is frequent in high-grade gliomas. Cancer Res 55: 1941–1945 7728764

[bib17] Pines J, Hunter T (1989) Isolation of a human cyclin cDNA: evidence for cyclin mRNA and protein regulation in the cell cycle and interaction with p34cdc2. Cell 58(5): 833–846 2570636 10.1016/0092-8674(89)90936-7

[bib18] Ritzi M, Baack M, Musahl C, Romanowski P, Laskey RA, Knippers R (1998) Human minichromosome maintenance proteins and human origin recognition complex 2 protein on chromatin. J Biol Chem 273(38): 24543–24549 9733749 10.1074/jbc.273.38.24543

[bib19] Rolfe KJ, Crow JC, Benjamin E, Reid WMN, Maclean AB, Perrett CW (2001) Cyclin D1 and retinoblastoma protein in vulvar cancer and adjacent lesions. Int J Gynecol Cancer 11: 381–386 11737469 10.1046/j.1525-1438.2001.01039.x

[bib20] Scully RE, Boniglio TA, Kurman RT, Silverberg SG, Wilkinson EJ (Eds) (1994) Vulva. Epithelial tumors and related lesions. In Histological Typing of Female Genital Tract Tumors, pp. 64. Berlin/Heidelberg: Springer-Verlag

[bib21] Taylor WR, Stark GR (2001) Regulation of the G2/M transition by p53. Oncogene 20(15): 1803–1815 11313928 10.1038/sj.onc.1204252

[bib22] van Beurden M, de Craen AJM, de Vet HCW, Blaauwgeers JLG, Drillenburg P, Gallee MPW, de Kraker NW, Lammes FB, ten Kate FJW (1999) The contribution of MIB 1 in the accurate grading of vulvar intraepithelial neoplasia. J Clin Pathol 52: 820–824 10690171 10.1136/jcp.52.11.820 PMC501593

[bib23] van Beurden M, ten Kate F J, Smits HL, Berkhout RJ, de Craen AJ, van der Vange N, Lammes FB, ter Schegget J (1995) Multifocal vulvar intraepithelial neoplasia grade III and multicentric lower genital tract neoplasia is associated with transcriptionally active human papillomavirus. Cancer 75: 2879–2884 7773937 10.1002/1097-0142(19950615)75:12<2879::aid-cncr2820751214>3.0.co;2-w

[bib24] van Beurden M, van der Vange N, ten Kate FJW, de Craen AJ, Schilthuis MS, Lammes FB (1998) Restricted surgical management of vulvar intraepithelial neoplasia 3: focus on exclusion of invasion and on relief of symptoms. Int J Gynecol Cancer 8: 73–77 11576286 10.1046/j.1525-1438.1998.09733.x

[bib25] Van den Brule AJC, Meijer CJLM, Bakels V, Kenemans P, Walboomers JMM (1990) Rapid human papillomavirus detection in cervical scrapes by combined general primers mediated and type specific polymerase chain reaction. J Clin Microbiol 28: 2739–2743 2177751 10.1128/jcm.28.12.2739-2743.1990 PMC268265

[bib26] van Hoeven KH, Kovatich AJ (1996) Immunohistochemical staining for proliferating cell nuclear antigen, BCL2 and Ki-67 in vulvar tissues. Int J Gynecol Pathol 15(1): 10–16 8852440 10.1097/00004347-199601000-00002

[bib27] Viallard JF, Lacombe F, Belloc F, Pellegrin JL, Reiffers J (2001) Molecular mechanisms controlling the cell cycle: fundamental aspects and implications for oncology. Cancer Radiother 5(2): 109–129 11355576 10.1016/s1278-3218(01)00087-7

[bib28] Wilkinson EJ, Kneale B, Lynch PJ (1986) Report of the ISSVD Terminology Committee. Proceedings of the Eighth World Congress of the International Society for the Study of Vulvar Diseases. Mariehamn, Finland, September 9–15, 1986. J Reprod Med 31: 973 3772896

[bib29] Williams GH, Romanowski P, Morris LS, Madine M, Mills AD, Stoeber K, Marr J, Laskey RA, Coleman N (1998) Improved cervical smear assessment using antibodies against proteins that regulate DNA replication. Proc Natl Acad Sci USA 95: 14932–14937 9843993 10.1073/pnas.95.25.14932 PMC24553

[bib30] Wong MH, Saam JR, Stappenbeck TS, Rexer CH, Gordon JI (2000) Genetic mosaic analysis based on Cre recombinase and navigated laser capture microdissection. Proc Natl Acad Sci USA 97(23): 12601–12606 11050178 10.1073/pnas.230237997 PMC18810

[bib31] Zamparelli A, Masciullo MV, Bovicelli A, Santini D, Ferrandina G, Minimo C, Terzano P, Costa S, Cinti C, Ceccarelli C, Mancuso S, Scambia G, Bovicelli L, Giordano A (2001) Expression of cell-cycle-associated proteins pRB2/p130 and p27kip in vulvar squamous cell carcinomas. Hum Pathol 32(1): 4–9 11172288 10.1053/hupa.2001.20371

[bib32] Zur Hausen H (2002) Papillomaviruses and cancer: from basic studies to clinical application. Nat Rev Cancer 2: 342–350 12044010 10.1038/nrc798

